# *Archolaemus janeae* (Gymnotiformes, Teleostei): First insights into karyotype and repetitive DNA distribution in two populations of the Amazon

**DOI:** 10.1002/ece3.8092

**Published:** 2021-11-09

**Authors:** Paula Pinto Rodrigues, Milla de Andrade Machado, Ananda Marques Pety, Danillo dos Santos Silva, Augusto Cesar Paes de Souza, Julio Cesar Pieczarka, Cleusa Yoshiko Nagamachi

**Affiliations:** ^1^ Laboratório de Citogenética Centro de Estudos Avançados da Biodiversidade Instituto de Ciências Biológicas Universidade Federal do Pará (UFPA) Belém Brazil; ^2^ Universidade do Estado do Pará Belém Brazil; ^3^ Laboratório de Estudos da Ictiofauna da Amazônia Instituto Federal do Pará Abaetetuba Brazil

**Keywords:** Amazon basin, FISH, Glass knifefish, karyotypes, Neotropical

## Abstract

*Archolaemus*, one of the five genera of Neotropical freshwater fish of the family Sternopygidae (Gymnotiformes), was long considered a monotypic genus represented by *Archolaemus blax*. Currently, it consists of six species, most of them occurring in the Amazon region. There are no cytogenetic data for species of this genus. In the present study, we used classical cytogenetics (conventional staining and C‐banding) and molecular cytogenetics (probes of telomeric sequences and multigenic families 18S rDNA, 5S rDNA, and U2 snDNA) to study the karyotype of *Archolaemus janeae* from Xingu and Tapajós rivers in the state of Pará (Brazil). The results showed that the two populations have identical karyotypes with 46 chromosomes: four submetacentric and 42 acrocentric (2*n* = 46; 4m/sm + 42a). Constitutive heterochromatin occurs in the centromeric region of all chromosomes, in addition to small bands in the interstitial and distal regions of some pairs. The 18S rDNA occurs in the distal region of the short arm of pair 2; the 5S rDNA occurs in five chromosome pairs; and the U2 snDNA sequence occurs in chromosome pairs 3, 6, and 13. No interstitial telomeric sequence was observed. These results show karyotypic similarity between the studied populations suggesting the existence of a single species and are of great importance as a reference for future cytotaxonomic studies of the genus.

## Abstract 2

In this article, we describe for the first time the karyotype of *Archolaemus janeae* from Xingu and Tocantins rivers, Brazilian Amazon region, relating these cytogenetic data to their geographic distribution and to the cytogenetic data already available in the literature for the Sternopygidae family.

## 1 INTRODUCTION

Gymnotiformes, a diverse order of Neotropical electric fish, has more than 250 species widely distributed in Central and South America, with great diversity and abundance in the Amazon Basin (Albert, 2001; Albert & Crampton, 2005; Ferraris et al., 2017; Fricke et al., 2021).

Among the five families of the order, Sternopygidae comprises six genera: *Sternopygus* Müller, Troschell, 1846, *Eigenmannia* Jordan & Evermann, 1896, *Rhabdolichops* Eigenmann, Allen, 1942, *Distocyclus* Mago‐Leccia, 1978, *Archolaemus* Korring, 1970, and *Japigny* Meunier, Jégu, Keith, 2011 (Albert, 2001; Ferraris et al., 2017; Meunier et al., 2011). The genus *Archolaemus*, proposed by Korringa (1970), was initially considered monotypic, being represented by a sole species, *Archolaemus blax*, with distribution along the Araguari, Branco, Tapajos, Tocantins, and Xingu rivers (Vari et al., 2012).

Based on morphological analysis, Vari et al. (2012) consider that *Archolaemus* comprises six species, of which five (*A*. *blax*, *Archolaemus ferreirai*, *Archolaemus janeae*, *Archolaemus luciae*, and *Archolaemus santosi*; Vari et al., 2012) are distributed in the Amazon Basin and its tributaries and one (*Archolaemus orientalis* Stewart, Vari et al., 2012) occurs in the São Francisco River basin of eastern Brazil. To date, cytogenetic study for this genus is still lacking.

The cytogenetic studies published for Gymnotiformes have given insight into the diversity and karyotype evolution of this order (Cardoso et al., 2015; Fernandes, Baumgärtner, et al., 2017; Fernandes, Paiz, et al., 2017; Faria‐Pereira et al., 2019; Milhomem et al., 2012; Sene et al., 2014; Silva et al., 2008; da Silva et al., 2016; da Silva et al., 2014). The diploid chromosome number (2*n*) ranges from 2*n* = 24 in *Apteronotus albifrons* (Linnaeus, 1766) (Apteronotidae; Fernandes, Paiz, et al., 2017; Takagui et al., 2017) to 2*n* = 74 in *Rhabdolichops* cf *eastwardi* Lundberg, Mago‐Leccia, 1986 (Sternopygidae; Suárez et al., 2017). Chromosomal studies have shown that species diversity may be higher than previously considered, for there are morphologically similar species (cryptic species) with different karyotypes, such as in *Gymnotus carapo* Linnaeus, 1758 (Milhomem et al., 2008; Nagamachi et al., 2010).

In the Sternopygidae family, there are cytogenetic data for the genera *Eigenmannia*, *Sternopygus*, and *Rhabdolichops*, with genus *Eigenmannia* being the most studied. *Eigenmannia* species have 2*n* ranging from 28 to 46 chromosomes and varied sex chromosome systems (Almeida‐Toledo et al., 1985, 2001; Fernandes et al., 2010; Henning et al., 2008; Sene et al., 2014; Silva et al., 2009). Cytogenetic data for genus *Rhabdolichops* are available for the species *R*. *troscheli* (Kaup, 1856) with 2*n* = 54 and *R*. cf. *eastwardi* with 2*n* = 74 (Suárez et al., 2017). *Sternopygus macrurus* (Bloch, Schneider, 1801) is the only species of genus with cytogenetic data available, has a 2*n* = 46, but presents different karyotypic composition for specimens from different hydrographic basins (Almeida‐Toledo et al., 1993; Fernandes, Baumgärtner, et al., 2017; Fernandes, Paiz, et al., 2017; Silva et al., 2008).

In this article, we report for the first time the karyotype of *Archolaemus janeae* from Xingu and Tapajos rivers (both in Pará state), Brazilian Amazon. As *A*. *janeae* is originally known from populations in these rivers (e.g., Vari et al., 2012), we compared the karyotypes found in both rivers to test the previous hypothesis that those populations belong to a single species. We also relate the cytogenetic data to those previously reported for Sternopygidae.

## 2 MATERIAL AND METHODS

### 2.1 Samples

**FIGURE 1:**
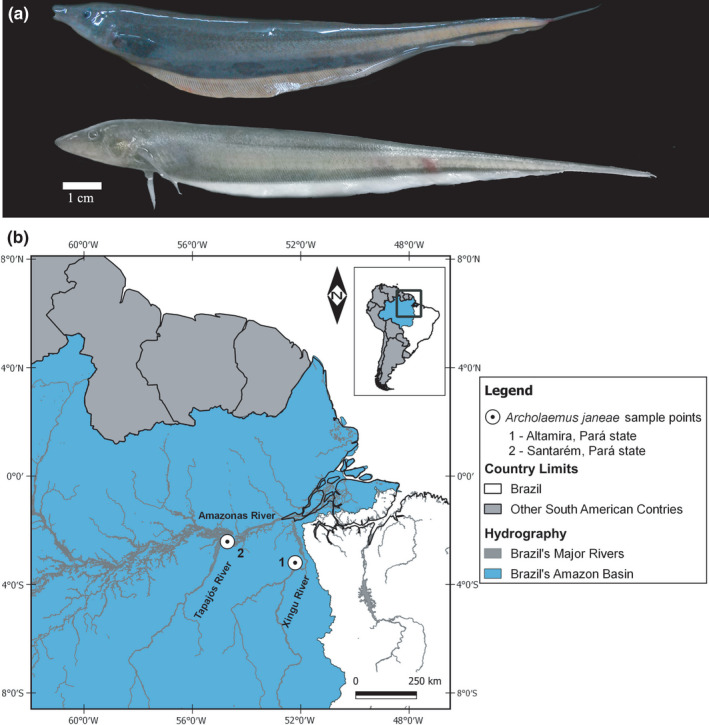
Specimens of *Archolaemus janeae* (a) from locality 1 (above) and locality 2 below; map showing the collection locations of the samples (b). The map was made using QGIS v. 3.10.7. The shape files containing country limits and hydrography were obtained from DIVA‐GIS [Hijmans et al., 2004], https://www.diva‐gis.org/gdata, and from the Agência Nacional de Águas, Superintendência de Planejamento de Recursos Hídricos https://dadosabertos.ana.gov.br/datasets/b78ea64219b9498c8125cdef390715b7_0

About the sample of *Archolaemus janeae* (Figure 1) used in the present study (Table 1), six specimens were collected in the Xingu River, municipality of Altamira, Pará, Brazil. The specimens were collected with the aid of an electric discharge detector and nylon handles. The IBAMA (Brazilian Institute of the Environment and Renewable Natural Resources) provided the seven specimens collected in the Tapajos River, Santarém, Pará, Brazil. JCP has a permanent field permit number 13,248 from “Instituto Chico Mendes de Conservação da Biodiversidade.” The Cytogenetics Laboratory of UFPa has permit number 19/2003 from the Ministry of Environment for sample transport and permit 52/2003 for using the samples for research. The Ethics Committee of the Federal University of Para (Comitê de Ética Animal da Universidade Federal do Pará) approved this research (Permit 68/2015).

**TABLE 1:** Specimens of *Archolaemus janeae* species analyzed

Species	Locality	Samples	Coordinates	Protocol number
*Archolaemus janeae*	Xingu River Altamira‐PA	6 (3♂ and 3 undetermined)	3°11′41″S/52°12′33″W	P‐2402; P‐2409; P‐2436; P‐2452; P‐2457; P‐2466
	Tapajós River Santarém‐PA	7 (2♂3 ♀ and 2 undetermined)	2°24′52″S/54°42′36″W	P‐4139; P‐4140; P‐4141; P‐4142; P‐4143; P‐4144; P‐4145

The samples from Xingu River were identified at the Museu Paraense Emílio Goeldi (MPEG), where they are deposited. The samples from the Tapajós River have been deposited in the Ichthyological Collection of the Center for Advanced Biodiversity Studies (CEABIO), Federal University of Pará.

### 2.2 Cytogenetic analysis

Metaphasic chromosomes were obtained by fermentation‐based induction of mitosis (Bertollo, 1986) followed by direct chromosome extraction (Bertollo et al., 1978). The specimens were euthanized with eugenol (Fernandes et al., 2016). The constitutive heterochromatin (CH) was detected using C‐banding technique (Sumner, 1972). Fluorescence in situ hybridization (FISH) (Pinkel et al., 1986) was performed with probes for the 18S rDNA (Table 2), 5S rDNA (Table 3), U2 snRNA (Table 4), and telomeric sequence (TTAGGG)*n* (Table 5). Probes were labeled by PCR‐based incorporation of biotinylated dUTP (Invitrogen) or nick translation with a BioNick kit (Invitrogen) for biotin staining, and with a Dig‐Nick kit (Roche) for digoxigenin staining. Images were captured using a Nikon H550S Fluorescence Photomicroscope equipped with the Nis‐Elements Software, and the karyotypes were organized using Adobe Photoshop CC2018. Chromosomes were classified into two groups, metacentric/submetacentric (m/sm) and subtelocentric/acrocentric (st/a), and arranged in decreasing order of size for each group (adapted from measurements in Levan et al., 1964).

**TABLE 2:** Primer sequences used in this study to amplify the 18S rDNA

Sequence repeat	Primer sequences (5′–3′)	Reference
18S rDNA	F CCG CTT TGG TGA CTC TTG AT	Hatanaka and Galetti (2004)
	R CCG AGG ACC TCA CTA AAC CA	

Thermal profile			
Stage	Time	Temperature	Cycles
1	5 min	95°C	
2	1 min	95°C	2–4, 30×
3	30 s	50°C	
4	45 s	72°C	
5	5 min	72°C	

**TABLE 3:** Primer sequences used in this study to amplify the 5S rDNA

Sequence repeat	Primer sequences (5′–3′)	Reference
5S rDNA	F GCCACACCACCCTGAACAC	Suárez et al. (2017)
	R GCCTACGACACCTGGTATTC	

Thermal profile			
Stage	Time	Temperature	Cycles
1	4 min	95°C	
2	1 min	95°C	2–4, 35×
3	1 min	60°C	
4	2 min	74°C	
5	5 min	74°C	

**TABLE 4:** Primer sequences used in this study to amplify the U2 snRNA

Sequence repeat	Primer sequences (5′–3′)	Reference
U2 snDNA	F TCTCGGCCTATATTGGCTAA	Colgan et al. (1998)
	R GACGGTAGCTGCAATACCGG	

Thermal profile			
Stage	Time	Temperature	Cycles
1	4 min	95°C	
2	1 min	95°C	2–4, 30×
3	1 min	60°C	
4	2 min	74°C	
5	5 min	74°C	

**TABLE 5:** Primer sequences used in this study to amplify the telomeric sequence

Sequence repeat	Primer sequences (5′–3′)	Reference
Telomeric	F TTAGGGn	Ijdo et al. (1991)
	R CCCTAAn	

Thermal profile			
Stage	Time	Temperature	Cycles
1	5 min	94°C	
2	1 min	94°C	2–4, 35×
3	30 s	60°C	
4	1 min 30 s	72°C	
5	5 min	72°C	

## 3 RESULTS

**FIGURE 2:**
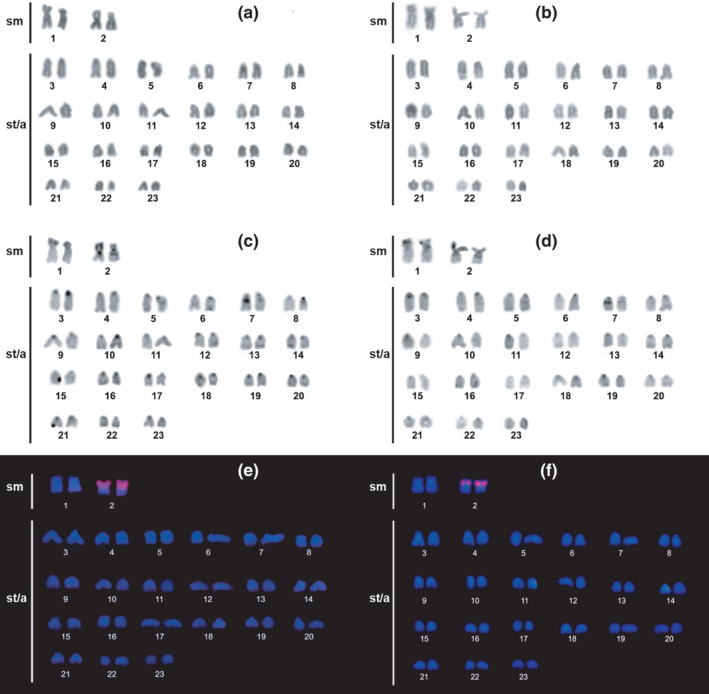
Karyotype of *Archolaemus janeae*: conventional staining (a, b); C‐banding (c, d); 18S rDNA probe (e, f). Tapajos sample (a, c, e); Xingu sample (b, d, f)

The specimens of *Archolaemus janeae* from the Xingu and Tapajos rivers have the same karyotype, with no difference in any of the markers used. The species has 46 chromosomes (Figure 2a, Tapajos sample; 2b, Xingu sample) and a karyotype formula composed by four bi‐armed and 42 acrocentric chromosomes (2*n* = 46, 4m/sm + 42a), without a cytogenetically visible sex system.

Constitutive heterochromatin (CH) occurs in the centromeric regions of all chromosomes in addition to terminal and interstitial signals in some chromosomes (Figure 2c, Tapajos sample; 2d, Xingu sample).

The 18S rDNA probe (Figure 2e, Tapajos sample; 2F, Xingu sample) hybridizes on the short arm of pair 2 evidencing the nucleolar organizing region (NOR), which here shows size heteromorphism.

**FIGURE 3:**
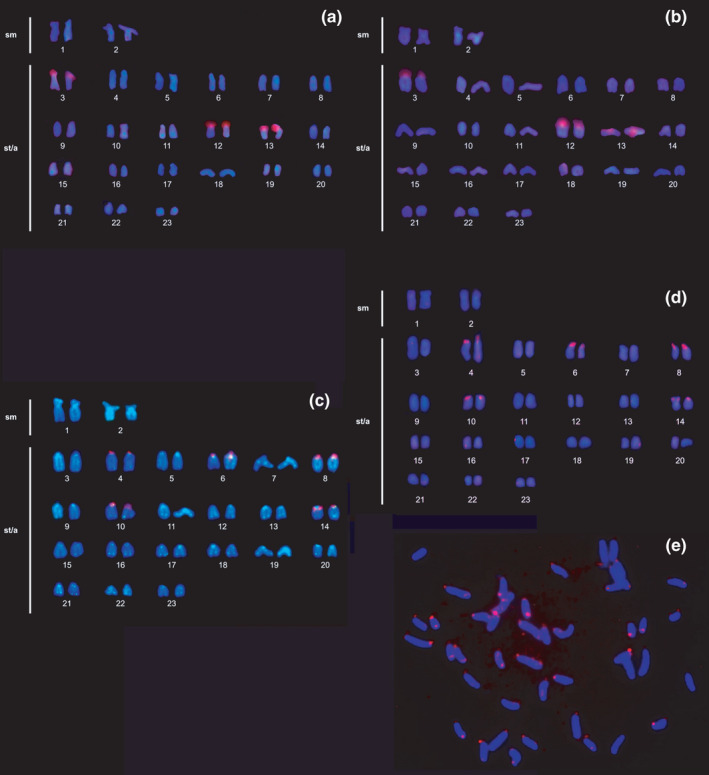
Karyotype of *Archolaemus janeae*. FISH with U2 snDNA probe in (a) Tapajos and (b) Xingu samples. (c) Double FISH with telomeric probe (green), and 5S rDNA probe (red) in Tapajos sample. (d) FISH with 5S rDNA probe and (e) telomeric probe in Xingu sample

The probe for the U2 snDNA sequence hybridizes on three chromosomal pairs: 3, 12, and 13 both in the Tapajos (Figure 3a) and in the Xingu (Figure 3b) samples. FISH with 5S rDNA sequence shows hybridization in five chromosomal pairs: 4, 6, 8, 10, and 14 in both samples (Figure 3c Tapajos; 3d Xingu). The telomeric probe (TTAGGG)*n* shows no evidence of the presence of interstitial telomeric sequences (ITS) (Figure 3c, Tapajos and Figure S1; 3e, Xingu).

## 4 DISCUSSION

*Archolaemus janeae* samples from both localities studied present the same karyotype (2*n* = 46, 4m/sm + 42st/a) being similar for all the markers used, despite being in different rivers (Table 1 and Figure 1).

A simple NOR signal is found in all but one species of the Sternopygidae family, although the location of the NOR can vary between different species and populations (Almeida‐Toledo et al., 2001; Araya‐Jaime et al., 2017; Fernandes et al., 2010; Fernandes et al., 2020; Sene et al., 2014; Silva et al., 2008, 2009; Suárez et al., 2017). Thus, a simple NOR may be a plesiomorphic characteristic for the genome of Sternopygidae representatives (Fernandes et al., 2010; Fernandes, Paiz, et al., 2017; Fernandes et al., 2020). Heteromorphism in the size of the NOR adjacent to a heterochromatic block has also been described for two other genera of family Sternopygidae: *Eigenmannia* (Silva et al., 2009, 2015) and *Sternopygus* (Fernandes, Baumgärtner, et al., 2017; Silva et al., 2008). This may be a consequence of tandem duplication of ribosomal genes, sister chromatid exchange, ectopic recombination, or unequal crossing over during meiosis (Baicharoen et al., 2016; Eickbush & Eickbush, 2007; Moreira‐Filho et al., 1984; Silva et al., 2008).

The multiple 5S rDNA sites found in *Archolaemus janeae* (Figure 3c,d) have been described for other species of the Sternopygidae family, such as *S*. *macrurus* (Fernandes, Baumgärtner, et al., 2017) and species of *Eigenmannia* (Araya‐Jaime et al., 2017; Fernandes et al., 2019; Sene et al., 2014). The 5S clusters are considered hot spots for chromosome break (Glugoski et al., 2018). These findings have been correlated with the insertion of transposable elements (TEs) into nontranscribed (NTS) 5S rDNA sequences, as observed in other fish genomes (Cioffi et al., 2010; Merlo et al., 2013; Silva et al., 2016). The association of this nontranscribed spacer with transposable elements may be responsible for the dynamics of the 5S rDNA sequence in the Gymnotiformes genome (Silva et al., 2016; Sene et al., 2014; Araya‐Jaime et al., 2017; Fernandes, Baumgärtner, et al., 2017; Fernandes, Paiz, et al., 2017; Fernandes et al., 2020), for it may establish a breakpoint region susceptible to chromosome breakage, nonhomologous recombination, and Robertsonian (RB) fusion (Glugoski et al., 2018). Alternatively, the variation in the number of sites for 5S can be explained by its presence close to fragile sites (Barros et al., 2017) and evolutionary breakpoint regions (Deon et al., 2020), leading to homologous and nonhomologous repair mechanisms such as Robertsonian fusions (Barros et al., 2017). In a study of *Eigenmannia* aff. *trilineata*, the 5S rDNA was found colocated with the snDNA U2 cluster (Araya‐Jaime et al., 2017). In *Archolaemus janeae*, however, as well as in *Gymnotus sylvius*, *Gymnotus inaequilabiatus*, *Gymnotus pantanal*, *Gymnotus javari*, *Gymnotus carapo*, and *Gymnotus pantherinus* (Utsunomia et al., 2014), colocation of these sequences is not seen. The cluster number appears to be conserved in six species of *Gymnotus*, and only one has multiple sites (Utsunomia et al., 2014). Studies using U2 snRNA are still scarce for fish, especially for order Gymnotiformes.

To date, cytogenetic information has been available for species from three of the six genera of Sternopygidae. Among them, *Eigenmannia* has been the most studied: A high karyotype diversity is seen between species of this genus, with 2*n* ranging from 28 to 46, and several examples of differentiated sex chromosome systems have been reported (for a review, see Araya‐Jaime et al., 2017; Fernandes et al., 2020; Sene et al., 2014; Silva et al., 2009, 2015). It has been proposed that populations of *Eigenmannia* experience isolation processes due to genetic drift caused by their low mobility and population density rates and that this facilitates the rapid fixation of structural or numerical chromosomal rearrangements (Araya‐Jaime et al., 2017; Silva et al., 2009). *Sternopygus macrurus* has a relatively more conserved karyotype (2*n* = 46, 30m/16sm; Silva et al., 2008); no difference in karyotype was found between specimens from several locations in the Amazon basin, but some differences in the karyotype formula were found between samples from São Francisco River (32m/14sm) and the Parana River (28m/18sm) described by Almeida‐Toledo et al. (1993). In contrast, *Rhabdolichops* was reported to have high karyotype variability, with 2*n* ranging from 54 to 74 (Suárez et al., 2017). The data presented here for *Archolaemus* demonstrate a 2*n* = 46 conserved karyotype. As both sampled rivers make up most of the geographic distribution of the genus, more studies on *Archolaemus* cytogenetics probably will show the same karyotype. Although the 2*n* = 46 for *Archolaemus* is present in *Sternopygus* and *Eigenmannia*, the karyotype composition differs, showing the occurrence of intrachromosomal rearrangements.

**FIGURE 4:**
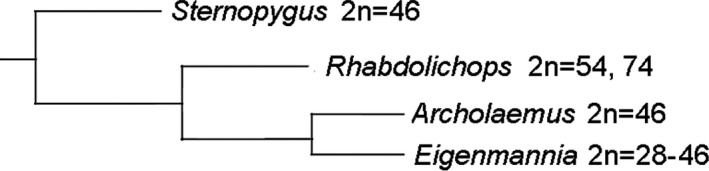
Phylogeny of the Sternopygidae genera for which cytogenetic data exist, based on Tagliacollo et al. (2016). The 2n found in the genera are reported

As we had the diploid numbers of each genus in the molecular phylogeny published by Tagliacollo et al. (2016) for Sternopygidae (Figure 4), it can be seen that 2*n* = 46 is present in three of the four genera analyzed and distributed throughout the phylogeny, suggesting that the 2*n* = 46 may be ancestral for this family and that chromosomal rearrangements have occurred in each genus.

## 5 CONCLUSION

Samples of *Archolaemus janeae* collected in the two major rivers of its occurrence show the same karyotype, suggesting that this species has karyotype stability. A comparison with karyotypes previously described for other species of Sternopygidae shows that the different genera have experienced different chromosomal evolution processes, with karyotypes preserved in some and variables in others. However, from the four genera in this family, three present 2*n* = 46. This suggests that a 2*n* = 46 may be ancestral to the family.

## CONFLICT OF INTEREST

The authors declare that there is no conflict of interest.

## AUTHOR CONTRIBUTIONS

**Paula Pinto Rodrigues:** Conceptualization (equal); data curation (equal); formal analysis (equal); investigation (equal); methodology (equal); visualization (equal); writing—original draft (equal); writing—review and editing (equal). **Milla de Andrade Machado:** Data curation (equal); formal analysis (equal); investigation (equal); methodology (equal); visualization (equal); writing—review and editing (equal). **Ananda Marques Pety:** Data curation (equal); formal analysis (equal); investigation (equal); methodology (equal); visualization (equal); writing—review and editing (equal). **Danillo dos Santos Silva:** Data curation (equal); methodology (equal); writing—review and editing (equal). **Augusto Cesar Paes de Souza:** Data curation (equal); methodology (equal); writing—review and editing (equal). **Julio Cesar Pieczarka:** Formal analysis (equal); funding acquisition (equal); methodology (equal); resources (equal); visualization (equal); writing—review and editing (equal). **Cleusa Yoshiko Nagamachi:** Formal analysis (equal); funding acquisition (equal); methodology (equal); project administration (equal); resources (equal); supervision (equal); visualization (equal); writing—review and editing (equal).

## Supporting information

Figure S1

Supplementary Material

## Data Availability

All data used in this research are available in the article. The authors are available for any further explanation.
