# Supplementary figures and images for: Archolaemus janeae (Gymnotiformes, Teleostei): First insights into karyotype and repetitive DNA distribution in two populations of the Amazon
Source: Ecol Evol. 2021 Nov 9;11(22):15468–76. doi: 10.1002/ece3.8092 (PMC8601878; doi:10.1002/ece3.8092)

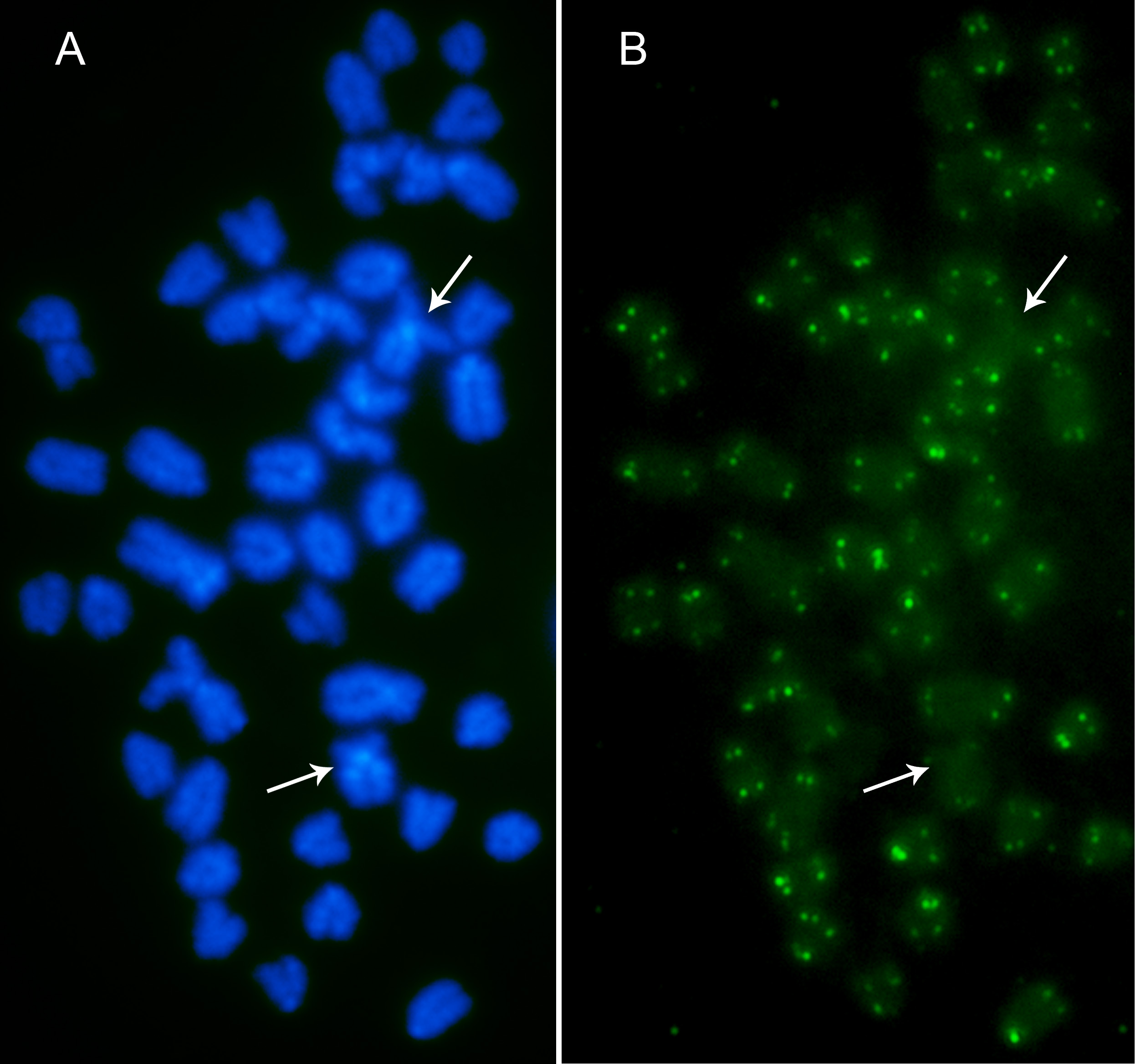

Supplement: Supplementary file 1 — Figure S1 [file ECE3-11-15468-s001.jpg]
