# Supplementary material for: Archolaemus janeae (Gymnotiformes, Teleostei): First insights into karyotype and repetitive DNA distribution in two populations of the Amazon
Source: Ecol Evol. 2021 Nov 9;11(22):15468–76. doi: 10.1002/ece3.8092 (PMC8601878; doi:10.1002/ece3.8092)
Supplement: Supplementary file 2 — Supplementary Material [file ECE3-11-15468-s002.docx]

**Legend of Supplementary Figure 1**

Figure S1- Metaphase corresponding to the karyotype in Figure 3C: A) DAPI banding; B) FISH with telomeric probes. Arrows: centromeric region of pair 2. Note that the bright signal at the centromere of pair 2 is not ITS as it might appear for its found in the DAPI band, but does not hybridize with telomeric probes.
